# A father’s legacy: the sperm epigenome, preimplantation development, and paternal environment

**DOI:** 10.1080/17501911.2025.2569301

**Published:** 2025-10-06

**Authors:** Leonard C. Steg, Isabelle M. Mansuy

**Affiliations:** a Laboratory of Neuroepigenetics, Brain Research Institute, Medical Faculty of the University of Zürich and Institute for Neuroscience, Department of Health Sciences and Technology of the ETH Zürich, Zürich, Switzerland; b Zürich Neuroscience Center, ETH and University of Zürich, Zürich, Switzerland

**Keywords:** Intergenerational epigenetic inheritance, sperm, epigenetics, preimplantation embryo, paternal effects, chromatin, RNA, environmental exposure

## Abstract

Paternal exposure to the environment can influence offspring phenotypes via a process known as intergenerational epigenetic inheritance. Such form of inheritance involves the sperm epigenome that is subjected to modifications by paternal exposure, which are carried from the father to the next generation. After fertilization, paternally inherited changes can manifest in the embryo and result in modified phenotypes later in life. To be long-lasting, these changes must either persist, escape the epigenetic reprogramming occurring after fertilization or be reinstated by guiding mechanisms during early development. This review discusses how the sperm epigenome instructs transcription and early embryonic development, and how environmental exposure can reshape this epigenetic information to influence developmental and transcriptional programs in the embryo. It addresses the patterns of penetrance in intergenerational epigenetic inheritance and considers how the sperm and embryonic epigenome can contribute to the variability of inherited phenotypes.

## Plain Language Summary

A growing number of studies suggest that a father’s environment such as his diet, stress level and other exposures, can influence the development and health of his offspring. These effects are thought to occur through epigenetic changes in sperm, meaning chemical modifications or molecules that do not alter the DNA sequence but can carry biological information. After fertilization, such changes can affect early embryo development and potentially contribute to differences in offspring traits. In this review, we summarize current research on how sperm can deliver epigenetic information to the embryo, how this influences early development, and why these effects vary across individuals and exposures. We also highlight the need for further studies to clarify how environmental signals are passed from sperm to embryo and how this process shapes the next generation.

## 1. Introduction

Heredity is a key biological process by which traits can be passed from one generation to the next. In species with sexual reproduction, transmission occurs via the germline and involves DNA in gametes. Historically, genetic material in sperm and oocytes has been regarded as the primary carrier of parental information thought to account for the inheritance of innate traits in the offspring. However, over the past two decades, growing evidence has challenged this view and suggested that non-genetic factors also play a role in heredity [1,2]. Thus, the health and physiology of an individual can be influenced by environmental factors and be reflected in its progeny [3,4]. This process, known as intergenerational epigenetic inheritance (IEI), depends on the germline and involves epigenetic changes induced by exposure in germ cells that can modify offspring’s phenotypes without altering the underlying DNA sequence.

Numerous examples of exposures with intergenerational effects on offspring development and health, including poor diet [5–9], stress [10–13], endocrine-disruptors [14,15], changes in the microbiome [16,17], infections or immune activation [18,19], but also beneficial conditions such as exercise [20], have been reported. The majority involve the paternal lineage but a few studies also reported inheritance via the matriline [2,21]. These findings challenge our understanding of heredity and are important for public health because they suggest broad effects of environmental exposures not only on the exposed generation but also its offspring. How environmentally induced changes to the sperm epigenome are transferred to the zygote and how this information is interpreted and established during early stages of embryonic development is not fully understood.

The major functions of the epigenome are to regulate genome activity, in some cases persistently and heritably without involving any change in the DNA sequence. Chromatin is a central component of the epigenome which consists of DNA wrapped around histone proteins that package the genome within the nucleus. DNA and histones are subjected to many modifications that can change their functions and structure. Major modifications include DNA methylation (DNAme) [22] that consists of the addition of a methyl group to cytosines, and histone post-translational modifications (PTMs) and incorporation of histone variants, which influence chromatin accessibility and genome activity [23,24]. Epigenetic marks are added, read or erased by various epigenetic enzymes and transcription factors (TFs) [23]. Further to epigenetic modifications, the genome also has a dynamic three-dimensional (3D) organization with open regions (euchromatin) supporting active transcription and condensed regions (heterochromatin) typically favoring repression. At a broad scale, transcriptionally active and inactive regions of the genome are partitioned into A and B compartments, respectively [25,26]. Finally, non-coding RNA also contributes to genome regulation by modulating transcription, RNA stability and chromatin states [27].

This review focuses on the transfer of epigenetic information from sperm to embryo and the consequences for preimplantation development in mammalian paternal models, primarily mice. It first describes the epigenome of mature sperm and the embryo during preimplantation development, then summarizes studies showing how the paternal epigenome can influence embryonic transcription and development under physiological conditions. It comprehensively examines how various paternal environmental exposures alter the sperm epigenome and how these changes affect the preimplantation embryo. It concludes with a discussion on the penetrance of IEI and the potential mechanisms of non-penetrant IEI in sperm and embryo development. The vast majority of studies discussed in this review are in mice or rats and studies involving other species are indicated as such.

## 2. The mature sperm epigenome

Male gametes in mammals derive from spermatogonial stem cells that differentiate into spermatocytes, spermatids then sperm during spermatogenesis. Spermatogenesis is a highly coordinated process that involves mitosis and two rounds of meiosis, accompanied by morphological changes of spermatogenic cells [28]. Once released from testes into the epididymis, a tubular organ attached to each testis, sperm continue to mature, become motile and gain the capacity to fertilize an oocyte [29]. During spermatogenesis, the sperm genome gets highly compacted, a process that is essential for fertility and DNA protection [30–32]. Compaction occurs via the replacement of most histones by protamines, which are small basic proteins. A fraction of histones is however retained, from 1% to 7.5% in mice [33–35] to about 10% in men [33,36]. The precise amount and genomic localization of retained histones remain debated, with some studies reporting retention at CpG-rich promoters [33–38] and others in gene-poor regions [39,40].

Histones retained in sperm carry PTMs [41] such as H3K4me3 at CpG-rich promoters [34,37,40,42], H3K27me3 at developmental gene promoters [33] or intergenic regions [42] and H3K9me3 at centromeric repeats [40]. Protamines also carry PTMs but the distribution and functional relevance of PTMs on histones and protamines remain largely unknown. Protamines PTMs may be important for sperm function such as motility and fertility [43–45]. Notably, a recent study challenged published work on mouse sperm chromatin and suggested that datasets are contaminated by signal from somatic cells’ chromatin. While this needs to be experimentally addressed, it highlights the need for careful interpretation of existing data and their validation [46]. Further to PTMs, the genome of male germ cells is also modified by DNAme during spermatogenesis. This involves *de novo* cytosine methyltransferases such as DNMT3 and results in global hypermethylation of mature sperm [37,47,48]. CpG-rich regions, such as CpG islands (CGIs), remain however mostly unmethylated at regulatory elements [47,49]. Mature sperm chromatin has a specific 3D organization that is different from that of somatic cells. While A/B compartments can be identified [35,50,51], it is unclear if topologically associating domains (TADs) also exist [35,46,50–52], even if structural proteins such as CTCF, cohesin and TFs are present in sperm nucleus [35,50,53,54]. Further, inter-chromosomal contacts are increased in sperm due to tight chromatin compaction [50,51].

Although sperm cells are thought to be transcriptionally silent, they contain rich RNA populations. These populations likely originate from transcription in prior spermatogenic cells during spermatogenesis and/or from RNA acquisition during epididymal maturation [55–57]. Small RNAs (sRNAs) have been the most studied and are modified during spermatogenesis and sperm maturation. During epididymal transit, extracellular vesicles from epididymal epithelial cells, known as epididymosomes, deliver sRNAs to sperm cells. Epididymosomes are particularly enriched in transfer RNA (tRNA)-derived RNA (tDRs, recommended standard ontology [58]) and microRNA (miRNA) [59,60]. Owing to epididymosomes-to-sperm communication, maturing sperm drastically alter their sRNA cargo and ultimately contain 65%–70% tDRs, 7%–17% miRNA and 5%–10% PIWI-interacting RNA (piRNA) and small amounts of mRNA [9,60–62]. However, not all sperm tDRs originate from epididymosomes and some, such as mitochondrial tDRs (mt-tDR), can derive from tRNAs produced by sperm itself during spermatogenesis [9]. Both coding and long non-coding RNA (lncRNA) have also been identified in mature sperm, mostly as fragmented transcripts and with only a few intact RNAs [55,63–66].

## 3. The paternal epigenome of the preimplantation embryo

After fertilization, the paternal and maternal epigenomes undergo extensive reprogramming during which most gamete-specific marks are erased. This results in a totipotent chromatin state required for the formation of the newly developing organism [67–70]. Embryonic preimplantation development starts from the zygote, which initially has two separate pronuclei each containing one of the parental genomes. After the merging of pronuclei, the zygote undergoes a series of cleavage divisions, first forming a 2-cell embryo that develops into a blastocyst. At the blastocyst stage, cells have begun to differentiate into the trophectoderm and inner cell mass which, respectively, give rise to extra-embryonic tissues and contain pluripotent cells that will form the embryo [71,72]. A critical milestone in preimplantation development is the maternal-to-zygotic transition, during which transcriptional control shifts from maternally-provided factors to the embryonic genome. This is mediated by two waves of zygotic genome activation (ZGA), a minor wave occurring during the middle-to-late 1-cell stage and a major wave at late 2-cell stage in mice [73–75]. Both waves of ZGA depend on transcriptional activity which implies that the paternal epigenome, which is in a repressed state in sperm, undergoes a broad reprogramming to be re-activated.

A notable step for the paternal genome is that it rapidly decondenses immediately after fertilization. This is accompanied by the removal of protamines and the incorporation of maternally derived histones, particularly the active variant H3.3, and involves the action of maternal factors such as SRPK1, NPM2 and HIRA [76–79]. It is not known if histones retained in sperm are maintained in the embryo or are exchanged with maternal histones. In addition to maternal histones, the paternal genome also receives a new set of histone PTMs, which initially are active histone PTMs followed by a delayed acquisition of repressive modifications [80–82]. These histone PTMs can have a non-canonical distribution and are not restricted to promoters i.e., H3K4me3 is broadly distributed over the genome [81,82]. Embryo-specific chromatin states are thought to facilitate genome reprogramming and ZGA (reviewed elsewhere [68,70]). It remains unclear if histones with or without their PTMs are directly inherited from sperm. While direct inheritance of sperm H3K4me3 has been proposed [83,84], the presence of H3K4me3 at similar genomic regions in the embryo may result from *de novo* deposition guided by DNAme [37,82]. Depending on the genomic context, histone PTMs can also guide the deposition of DNAme. For example, H3K9me3 was proposed to promote DNAme and heterochromatin formation at specific CpG-rich regions [85]. Further to histones, DNAme is globally remodeled on the paternal genome after fertilization. It is removed by a process actively catalyzed by maternal TET3 [86–88]. However, certain genomic elements, such as imprinting control regions (ICRs) and intracisternal A-particle (IAP) retrotransposons, are not demethylated and keep their paternal DNAme patterns [89,90]. Global DNAme level stays low throughout preimplantation development and increases again after implantation, mediated by DNMT3 *de novo* methyltransferases [91,92]. The 3D conformation of the paternal genome also undergoes major reorganization during preimplantation development [52,93]. Chromatin is initially in a relaxed state after protamine-to-histone exchange and gradually re-acquires higher-order features such as compartments, TADs and loops as development progresses. This is driven by TFs and structural proteins like CTCF and cohesin [52,53,93,94]. At the 8-cell stage, these factors help to integrate the paternal and maternal genomes into a unified architecture [53,93].

With respect to RNA, the vast majority in the zygote is maternally derived and comes from the oocyte [95]. However, RNA also comes from sperm and despite being in minute amounts compared to the oocyte, paternal RNA can influence gene expression in the zygote [9,60,96,97]. This suggests an important molecular route for paternal contribution to the offspring. The extent to which paternal sRNA contribute to the overall embryonic transcriptome remains however unkown, due to the difficulty in determining the parental origin of sRNA [98,99]. Further, during the first wave of ZGA, the paternal genome has higher transcriptional activity than the maternal genome [100,101]. This results in a rapid increase in paternal transcripts, that rise from a low level to about 25% of the total transcriptome in the early 2-cell embryo [95]. Thus, through the two waves of ZGA, the embryo gradually shifts from relying on transcripts derived from parental gametes to producing its own RNA by transcription from its maternal and paternal genome [95,102].

In sum, after fertilization and preimplantation development, the paternal epigenome undergoes rapid and extensive remodeling to enable the transition to totipotency and support early embryonic development. Yet, before the onset of ZGA, the embryo still relies on epigenetic information inherited from both parental germ cells that provides instructions about proper initiation of transcriptional programs for further development.

## 4. The instructive sperm epigenome

**Figure 1.**
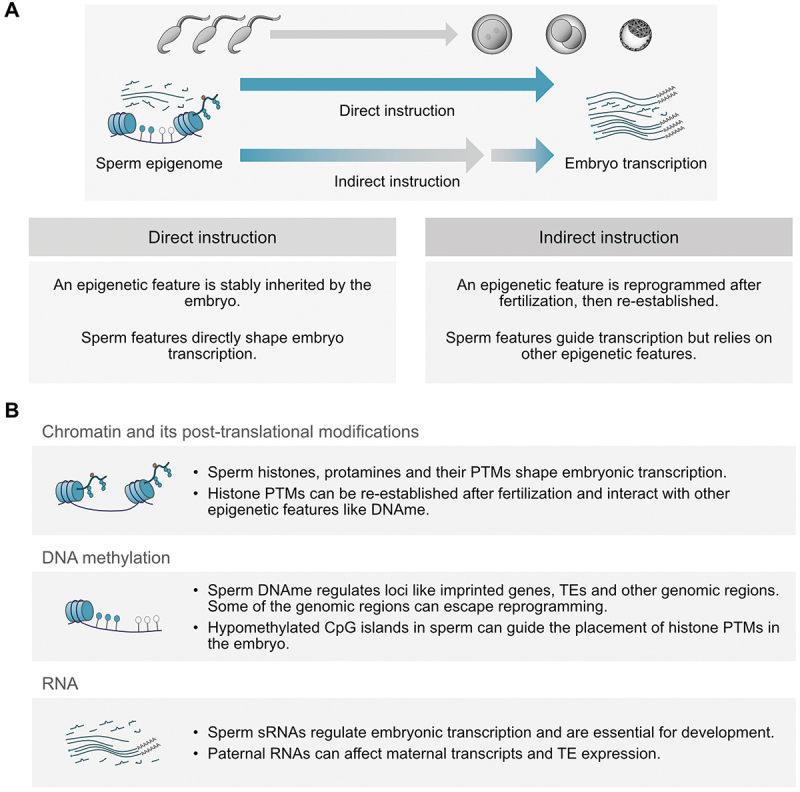
Paths of transcriptional instruction from haploid sperm to diploid embryo. (A) Schematic illustrating differences between direct and indirect instruction of epigenetic features from sperm to embryo (mouse sperm is drawn). Both mechanisms likely occur in parallel, with different epigenetic features using one of the pathways. (B) Overview of the potential of various sperm epigenetic features to guide transcriptional regulation in the early embryo.

Abbreviations: DNAme: DNA methylation, PTM: Post-translational modification, sRNA: small RNA, TE: Transposable element.

To influence development and transcription in the early embryo, sperm can provide epigenetic information that is directly inherited at fertilization. Importantly, not all epigenetic features from sperm are stably maintained after fertilization and some can be erased and subsequently reestablished during preimplantation development, while others may be lost entirely [68,82]. Despite such reprogramming, features of the sperm epigenome can be reflected in embryonic transcription, including regions undergoing reprogramming. This suggests inheritance of multiple layers of the epigenome, where non-reprogrammed elements may act as signals guiding the reestablishment of other epigenetic marks to eventually influence embryonic gene expression (Figure 1).

Several studies suggest that sperm histones and their PTMs can instruct transcriptional outcomes in the early embryo. For instance, increased histone retention due to disruption of poly (ADP-ribose) metabolism in sperm alters transcriptional activity in 2-cell embryos [103]. However, it is unknown if these effects are solely due to histone retention or also involve other epigenetic marks such as DNAme [104]. Further, overexpression of the histone demethylase KDM1A in spermatogonia, male germline stem cells, reduces H3K4me2 levels in mature sperm, which alters transcription at regions normally marked by H3K4me2 in 2-cell embryos [105]. KDM1A overexpression also affects H3K4me3, whose level is increased in sperm [42]. The altered regions are also enriched for H3K4me3 on the paternal genome in zygotes shortly before the first cleavage, and the enrichment persists throughout preimplantation stages. This was interpreted as full retention of H3K4me3 from sperm to embryo, but it may also reflect erasure of the marks shortly after sperm entry into the oocyte, followed by reestablishment at specific loci based on inherited DNAme patterns [37,106]. Additionally, germline-specific knockout of SCML2, which reduces H3K27me3 levels in sperm, leads to transcriptional dysregulation in embryos, particularly at genes critical for development [107].

Together, these studies highlight the functional relevance of sperm histone modifications in shaping the transcriptional profile of the early embryo. But further work is needed to determine if histone PTMs are directly transmitted and retained from sperm to zygote. This was shown in human embryos, where heterochromatic regions can be directly inherited from sperm, but not in mice [108]. Nonetheless, it provides evidence that mammals possess mechanisms for direct inheritance of chromatin marks. Further, sperm appear to carry histone proteins outside the nucleus, and while it is not known if these histones are integrated into the embryonic genome after fertilization, their depletion delays development, suggesting a functional role [109]. There is also evidence that the sperm histone-modifying complex PRC2 regulates embryonic expression of transposable elements (TEs). This suggests that epigenetic regulators in sperm can influence embryo transcription, possibly via sperm histone PTMs [110].

Sperm DNAme also plays a critical role in early development, most notably in the regulation of genomic imprinting. During gametogenesis, specific ICRs acquire DNAme marks that establish parent-of-origin-specific expression of imprinted genes in the early embryo [111,112]. These methylation marks are selectively established and protected from active or passive demethylation following fertilization and thus are faithfully inherited (see for review [113,114]). Their disruption can result in severe developmental abnormalities and imprinting disorders [115,116]. It is now clear that ICRs are not the only regions that can escape reprogramming [117]. Evolutionarily young TEs retain DNAme during global demethylation after fertilization [118]. This is likely a protective mechanism to block their activity and prevent their potential threat to genome integrity [119]. Proper regulation of TEs expression is crucial for preimplantation development, and both overexpression and insufficient activation have been linked to embryonic lethality and developmental defects [120,121]. An example of direct inheritance of sperm DNAme concerns the IAP retrotransposon family, which largely resists demethylation after fertilization [89,91,122]. Tight control over TEs is important because TEs not only regulate their own expression but can also function as regulatory elements that influence the expression of nearby genes [123–125].

While much attention has been given to regions that retain high levels of DNAme in sperm, loci with low levels of methylation also need to be considered. Despite the globally hypermethylated landscape of the sperm genome [47], CGIs are protected from methylation during spermatogenesis, primarily through histones, their PTMs and TF binding. This hypomethylated state can be maintained in the embryo. Lowly methylated CGIs have been shown to guide the placement of histone modifications in early development, notably H3K4me3, which is associated with transcriptionally permissive chromatin [37]. Hypomethylated regions may be further protected from *de novo* methylation after implantation by the binding of sequence-specific TFs [126].

Regarding sperm RNA, there is broad evidence that sRNAs acquired during spermatogenesis or epididymal transit are essential for normal embryonic development. Conditional knockout of miRNA biogenesis enzymes such as *Dgcr8*, *Dicer* and *Drosha* in the epididymis or germ cells alters gene expression and the development in preimplantation embryos [96,127], indicating a functional role for sperm miRNA in early development. Sperm miRNAs can directly modulate maternal transcripts in the zygote and influence early development. Injection of an inhibitor of the sperm-borne miRNA miR-34c for instance, alters the level of maternal transcripts [128]. Further to miRNA, sperm tDRs have also been implicated in early development. They contribute to the downregulation of murine endogenous retrovirus-L (MERVL) TE in preimplantation embryos [60] by regulating the expression of histone genes [129]. Further, a highly abundant sperm tDR derived from tRNA-Val-CAC was found to regulate the transcription and splicing of embryonic mRNA, and its suppression reduces the first cleavage rate and delays embryo development [130]. Notably, sperm deliver not only sRNA but also mRNA, which have been suggested to contribute to early embryonic processes and epigenetic regulation, although their exact functions remain unclear [65,131,132].

These studies demonstrate that the sperm epigenome plays an instructive role for both the transcriptome and epigenome of the preimplantation embryo. This is coordinated by an interplay of different epigenetic modalities of the mature sperm. This interplay is well illustrated by the regulation of TEs in the preimplantation embryo, which are simultaneously controlled by histone PTMs, DNAme and sRNA [60,110,118,129]. Further, the different epigenetic features interact with each other to allow the transfer of epigenetic information, exemplified by sperm DNAme patterns that direct the reestablishment of H3K4me at specific genomic regions [37]. Conversely, the binding of TFs can prevent certain loci from re-methylating during epigenetic reprogramming [126]. The capacity of the sperm epigenome to instruct embryonic development also provides a potential route for the transfer of environmentally induced epigenetic alterations to the next generation.

## 5. From paternal environments to the preimplantation embryo

The environment and diet of a father can influence the development and physiology of its offspring. This may be explained by molecular changes induced by exposure in the father before and/or at the time of conception and their transfer to the oocyte at fertilization. This implies that changes are present in the embryo during its earliest stages of development. Changes caused by paternal exposure can derive from sperm and modify the epigenome and gene expression in the embryo.

Various environmental factors have been reported to alter chromatin and the RNA cargo in sperm and affect the developing embryo (Table 1). A folate-deficient diet disrupts H3K4me3 pattern in sperm, an effect reflected in H3K4me3 and gene expression profile in 8-cell embryos [83]. Likewise, environmental toxicants such as the herbicide glufosinate-ammonium affect H3K4me3 and H3K27ac in sperm, and likely influence allele-specific expression in 4-cell embryos [133]. These findings suggest that changes in sperm histone PTMs induced by exposure can modify the transcription of genes differentially marked by histone PTMs in the embryo, implying an instructive role of histone PTMs in intergenerational transmission.

**Table 1.** Summary of studies investigating the effects of paternal exposure on preimplantation embryo development.

Study	Paternal exposure	Duration	Delay between exposure and mating/fertilization	Fertilization method	Assessed in sperm	Embryo stage	Assessed in embryo	Key findings in sperm to embryo instruction	Reference
Lismer (2021)	Folate deficient diet	11 weeks	Immediate	Natural breeding after pharmacological superovulation	H3K4me3	8-cell	H3K4me3 and mRNA	Altered H3K4me3 in sperm partially retained in embryos. Embryo gene expression changes overlap with sperm H3K4me3 at promoters.	[83]
Ma (2022)	Glufosinate-ammonium	5 weeks	Immediate	Natural breeding after pharmacological superovulation	H3K4me3 and H3K27ac	4-cell	mRNA	Sperm H3K4me and H3K27ac linked to allele-specific gene expression in embryos.	[133]
Sharma (2016)	Low protein diet	6 - 9 weeks	Immediate	in-vitro fertilization	small RNA	2-cell toBlastocyst, focus on 2-cell	mRNA	Downregulation of tDR-4:31-Gly-CCC-1-M4 targets and MERVL targets in 2-cell embryos from low-protein diet fathers.	[60]
Watkins (2017)	Low protein diet	7 - 9 weeks	Immediate	Natural breeding		Blastocyst	Targeted genes of AMPK signaling pathway	Reduced expression of genes in AMPK signaling pathway in blastocysts from low-protein diet fathers.	[136]
Dura (2024)	Low protein high sugar diet OR gut microbiota dysbiosis	6 - 7 weeks	Immediate	in-vitro fertilization	small RNA	Blastocyst	mRNA	Paternal low protein/high sugar diet induces developmental delay and altered expression of metabolic genes. Paternal microbiome dysbiosis altered expression of lineage regulators in blastocysts. Both exposures increase gene expression variability of phenotype-relevant genes.	[137]
Binder (2012)	High fat diet	10 weeks	Immediate	in-vitro fertilization		2-cell to Blastocyst	Developmental staging	Paternal high fat diet induces developmental delay in embryos.	[138]
Morgan (2024)	Low protein diet or western diet	8 - 24 weeks	Immediate	Natural breeding	total RNA	2-cell to Blastocyst	Developmental staging	Accelerated embryonic development and earlier cleavage timing following paternal dietary exposures.	[139]
Bernhardt (2021)	High fat diet	15 weeks	Immediate	Natural breeding		8-cell	mRNA	Upregulation of Gata6 and Samd4b in embryos from high-fat diet fathers.	[140]
Tomar (2024)	High fat diet	2 weeks	Immediate or 4 weeks	in-vitro fertilization	small RNA	2-cell	mRNA	Paternal mitochondrial tDRs are directly inherited at fertilization.	[9]
Gapp (2018)	Early life stress	2 weeks	2.5 months	Natural breeding after pharmacological superovulation	total RNA	Zygote	total RNA	Correlated changes in mRNAs and long non-coding RNAs between sperm and zygotes due to paternal early life stress.	[64]
Dickson (2018)	Chronic social instability stress	7 weeks	2 weeks	Natural breeding after pharmacological superovulation	miRNAs 34c, 449a, 375 and 152	2-cell to morula	miRNAs 449a and 34c	Consistent downregulation of miR-34c and miR-449a in sperm and embryo up to morula stage.	[141]
Gapp (2021)	Dexamethason injection	1 time	2 weeks	in-vitro fertilization	small RNA	2-cell	small RNA and mRNA	Downregulation of six tDRs in embryos, two overlapping with altered sperm small RNAs. Transcriptomic effects observed primarily in late 2-cell stage, mainly genes in developmental pathways.	[142]
Trigg (2021)	Acrylamide injections	5 days	Immediate, 3 days or 24 days.	in-vitro fertilization	small RNA andproteome	2-cell	total RNA	Altered sperm miRNAs regulate expression of target genes in embryos.	[145]

Abbreviations: AMPK: AMP-activated protein kinase, MERVL: Murine endogenous retrovirus-L, miRNA: micro RNA, mRNA: messenger RNA, tDR: tRNA-derived RNAs.

Further to histone PTMs, RNA can also be altered by paternal exposure in sperm and embryo. Low protein diet (LPD) modifies the level of sperm tDRs, specifically those derived from tRNA-Gly-GCC, and impacts MERVL target gene expression in 2-cell embryos [60]. This suggests that sRNAs are involved in regulating TE-driven transcription in early development. As MERVL and other TEs contribute to the regulation of chromatin accessibility and 3D conformation in the preimplantation embryo [134,135], sperm tDRs may have an instructive role for the configuration of the epigenome and embryo development. Such role was indeed confirmed by microinjection of synthetic tDR-4:31-Gly-CCC-1-M4 in zygotes, which led to the repression of MERVL targets [60]. Microinjection of sperm-derived tDRs from males exposed to 6-month high fat diet (HFD) alters the expression of metabolic pathway genes in 8-cell embryos and blastocysts, further linking sperm tDRs to metabolic programming in early development. This also has consequences for adult metabolic phenotypes, which in injected individuals, were similar to those induced by direct paternal diet [5]. Recently, inheritance of mitochondrial tDRs (mt-tDRs) from sperm to embryo was also described, highlighting the potential role for sperm mt-tDRs in regulating preimplantation development [9].

Metabolic pathways are consistently influenced by paternal diet. LPD affects gene expression and shifts transcription of AMPK signaling components in blastocysts [136]. Paternal low protein/high sugar diet alters gene expression in metabolic pathways and causes developmental delay during preimplantation, while increasing the variability of MERVL retrotransposons expression [137]. HFD was also shown to cause developmental delay during preimplantation stages [138]. However, in some cases, both undernutrition by LPD and overnutrition by a western diet, were found to accelerate embryonic development, resulting in shorter intervals between early cleavage divisions [139]. Such opposite effects of paternal diet on the pace of preimplantation development are not understood but may result from experimental differences, such as the fertilization method or timing and duration of the dietary insult (see Table 1). Indeed, while 15-week of HFD leads to upregulation of *Gata6* and *Samd4b* in 8-cell embryos, genes involved in adipocyte differentiation [140], 2-week treatment causes differential expression of genes involved in metabolic and oxidative phosphorylation pathways in the embryo [9]. The fact that paternal diet leads to differential expression of genes in metabolic pathways suggests that embryos can be primed for metabolic dysfunction as early as during preimplantation development.

Beyond diet, adverse life conditions also have long-term effects on sperm and offspring. Early life stress modifies RNA populations in sperm and zygotes with some overlapping transcriptional changes between the two [64]. Chronic social instability in adulthood reduces the level of miR-449a and miR-34c-5p in sperm of exposed males and in 2-cell to morula embryos [141]. An acute stress is indeed sufficient to alter RNA in sperm and embryo. A single injection of dexamethasone, a synthetic glucocorticoid that mimics cortisol action, was shown to modify RNA populations in sperm and 2-cell embryos, particularly two tDRs that are down-regulated in both, as well as several genes involved in development [142]. Sperm RNA has been causally linked to stress phenotypes in the offspring since microinjection of sperm total RNA from stressed males into zygotes recapitulates most behavioral and metabolic phenotypes in the resulting offspring. Small or long sperm RNA alone do not [11,64]. However, in some cases, miRNAs were associated with the transfer of stress features to the offspring. Microinjection of nine combined miRNAs, which are increased in sperm of males stressed in adulthood, reduces the expression of maternal transcripts of their target genes in 2-cell embryos and lowers blood corticosterone in the adult [12,143]. The functional link between these miRNAs in paternal sperm and blood corticosterone in the offspring is however not know.

Sperm sRNA and the offspring can also be affected by pollutants and chemicals. The miRNA mmu-miR-6909-5p is altered in sperm by exposure to concentrated ambient particulate matter and dysregulates developmental genes in 4-cell embryos when injected in zygotes [144]. Several specific miRNAs (about 15) are increased in sperm by the reproductive toxicant acrylamide and cause a modest decrease of the expression of their target genes in the embryo [145]. Other paternal exposures, such as antibiotics leading to gut microbiome dysbiosis, differentially alter genes involved in cell differentiation and developmental pathways in blastocysts, suggesting changes in lineage allocation between primitive ectoderm and epiblast [137]. Further, expression variability in the embryo was used as a readout to evaluate intergenerational responses to paternal dysbiosis or low protein/high sugar diet. This exemplified how paternal exposure can induce hypervariability in the expression of genes related to phenotypic variance [137].

Together, these studies highlight that a broad range and duration of paternal exposures, from diet and stress to toxicants and from an acute to 6-month long exposure, can modify epigenetic states and gene expression in preimplantation embryos (Table 1). Some exposures can lead to overlapping changes in sperm and embryos, with genes involved in developmental pathways often being differentially expressed. It is important to better understand how different duration of environmental exposures affect the preimplantation embryo, however this requires direct comparison within the same study as done in [145]. Currently, it is not possible to draw global conclusions from different studies using the same exposure with varying duration because they are too sparse. Various mechanisms have been implicated in mediating intergenerational effects including histone PTMs and different RNA species such as lncRNA, tDRs and miRNAs. Whether the vectors of transmission are specific to each exposure and duration or involve common mechanisms remains unknown. Most studies discussed here (Table 1) use dietary or stress-related paternal exposure, which does not reflect the full diversity of exposures known to elicit intergenerational effects. Histone PTMs in sperm have been causally linked to gene expression in preimplantation embryos and may mediate changes induced by paternal exposure [42,83,105,107]. While this supports a role for sperm histone marks in shaping early embryonic transcription, it is not known if the inheritance of histone PTMs is direct or the result of guided reestablishment after fertilization [37,146]. Sperm DNAme was also shown to be instructive for embryo transcription and numerous studies show that paternal exposure can persistently modify sperm DNAme patterns [8,13,147]. However, a direct link between exposure induced-DNAme changes in sperm and transcriptional changes in the preimplantation embryo has not been established.

Today, sperm RNA is the most plausible vector of transfer of the effects of exposure with a functional role in modulating gene expression and development in preimplantation embryos [5,60,148,149]. Gaining the necessary causal evidence was possible as RNA profiling in sperm and early embryos is technically feasible and easier than for epigenetic marks such as DNAme or histone PTMs. Moreover, the known direct interaction of sperm non-coding RNA with the maternal transcriptome in the zygote that dominates prior to ZGA makes it possible to determine its influence on early developmental processes. By contrast, modalities such as histone PTMs and DNAme require that the paternal genome becomes transcriptionally active to exert their influence on the embryo, thus potentially delaying their impact. It is unlikely that the effects of individual exposures are transmitted by a single epigenetic factor or mechanism. Instead, inheritance is likely mediated by the combined action of several layers of the paternal epigenome, which interact within sperm and during preimplantation development.

The mechanistic knowledge on epigenetic inheritance gained in the recent years benefited from the use of refined methodologies such as zygotic RNA microinjection and technological innovations in omics [11,150]. While zygotic microinjection is an exquisite method to assess causality, it should use physiological amounts of RNA to properly mimic what is normally delivered by sperm. However, many reported microinjection experiments have used large excess of RNA, introducing a bias that makes data difficult to interpret [148,151]. We envision that the method could be further exploited in the field for instance, to inject TFs or chromatin components (or their coding mRNA sequences), factors suggested to be involved in information transfer from sperm to offspring [152,153]. Further, refined approaches based on genetic engineering [154] may be valuable in future studies to overexpress RNA in early embryos or induce locus-specific histone PTMs or DNAme by combination with epigenetic editing tools [155].

## 6. Concepts, consequences and challenges

**Figure 2.**
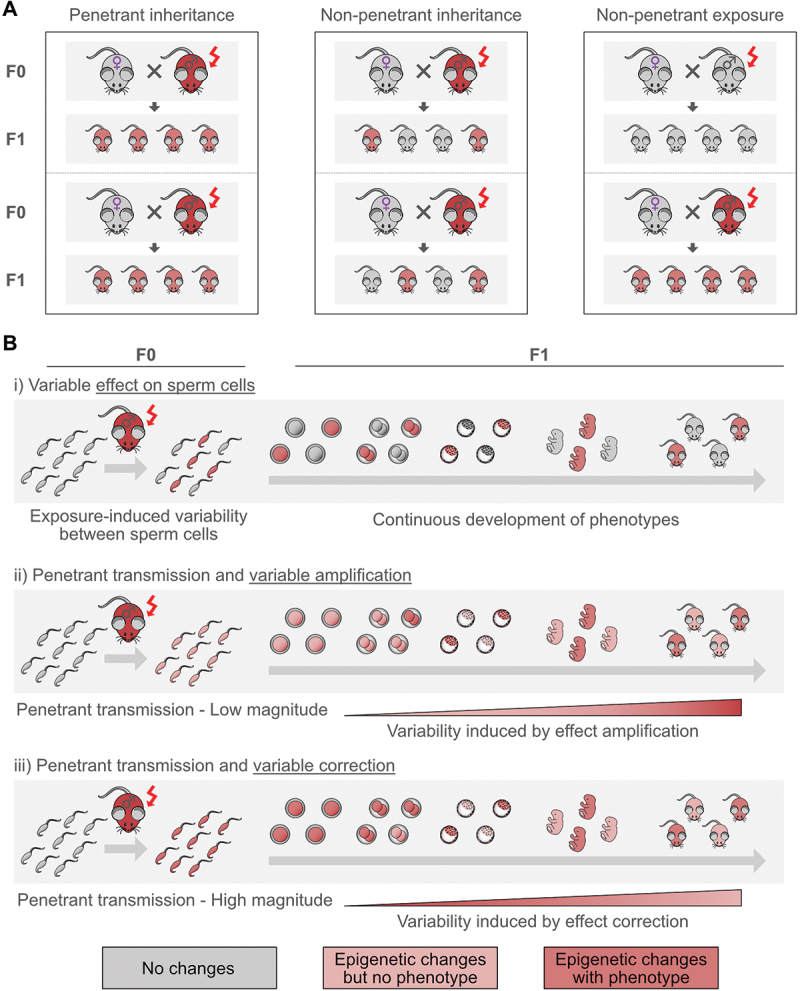
Modes of penetrance and mechanisms of phenotypic variability in intergenerational epigenetic inheritance. (A) Schematic representation of different penetrance patterns. *Penetrant inheritance* results in all offspring of an exposed individual displaying the altered phenotype. In *non-penetrant inheritance*, only a subset of the offspring is affected, despite uniform exposure. This must be distinguished from *non-penetrant exposures*, where only some offspring show phenotypic changes due to variability in how the exposure affected the F0 individual, rather than inheritance mechanisms. (B) Potential mechanisms underlying variability in offspring phenotypes. *i* sperm level variability. Only a subset of sperm carries epigenetic alterations due to exposure. Embryos derived from these sperm develop into phenotypically affected offspring. *ii* variability induced through effect amplification. All sperm carry mild alterations, but only a subset of embryos amplify these effects during development, leading to observable phenotypes. *iii* variability induced through effect correction. All sperm carry strong alterations, but only a subset of embryos correct these changes during development. Offspring that fail to correct the inherited alterations display the phenotype.

Several studies have reported cases of intergenerational effects of exposure in which only a subset of offspring from exposed fathers exhibit phenotypic alterations. Such pattern of partial penetrance is distinct from variability in paternal response and suggests that the effects of exposure may be propagated incompletely or selectively to the offspring. For instance, folate-deficient paternal diet leads to a spectrum of skeletal abnormalities in only some offspring [83], while ~30% of offspring from fathers exposed to 2-week HFD have altered metabolic phenotypes [9]. Similarly, paternal microbiome dysbiosis increases the risk of severe growth restriction in the offspring and eventually induces phenotypic alterations only in a subset of the animals [17]. In all these cases, exposure affects all fathers, but only some of their offspring manifest phenotypic traits, which has to be differentiated from a model where only a subset of exposed males is affected and transmit the phenotype (Figure 2(A)).

This concept raises important mechanistic questions about the timing and source of variability for such non-penetrant inheritance. One possibility is that variability arises at the level of sperm, with only some sperm cells carrying the epigenetic alterations necessary for phenotypic expression and each offspring derived from affected sperm have an altered phenotype (Figure 2(Bi)). Alternatively, all sperm cells may carry the altered information, but only a fraction of embryos or fetuses either amplify the effect above a certain threshold (Figure 2(Bii)) or only a subset activate correction mechanisms for the inherited changes (Figure 2(Biii)), resulting in selective manifestation of phenotypes. The possibility of early variability, either at sperm or zygote stage, has been supported by experimental data. RNA-sequencing of individual embryos from early 2-cell stage showed that 30% of embryos from HFD-exposed fathers have pronounced transcriptional changes relative to controls, a proportion closely matching the fraction of offspring with metabolic alterations [9]. Since embryos were assessed at a stage before initiation of major ZGA, this subgrouping was most likely derived from sperm or mechanisms happening shortly after fertilization.

The idea that epigenetic changes can influence gene expression variability has long been recognized. For instance, disruption of CTCF can destabilize enhancer–promoter interactions, leading to increased variability in gene expression [156,157]. In contrast, certain epigenetic features such as gene body DNAme, increased chromatin accessibility at promoters and certain histone PTMs such as H3K79 methylation have been shown to reduce expression variability [158–160]. These observations suggest that the epigenome influences not only how much a gene is expressed, but also how consistently it is expressed across cells. This ability to control expression variability could help explain why only some offspring are affected by environmental exposure, because certain epigenetic changes may create noise in gene regulation that influences whether a trait develops or not.

These models have profound implications for the design and interpretation of mechanistic studies of intergenerational inheritance. If only a subset of sperm carries epigenetic signals induced by exposure, then the effects in analyses may be diluted, changing the expected effect size and requiring adjusted statistics. To properly assess such variability in sperm, single-sperm resolution techniques are essential. Likewise, analyses of embryos or offspring must avoid pooling individuals, to not mask within-group variation that may have biological significance. Single-embryo and single-offspring analyses, paired with variability-focused approaches [137], will be critical to uncover meaningful mechanisms of non-penetrant epigenetic inheritance. Moving forward, which environmental exposures induce penetrant versus non-penetrant effects should be clarified because failing to distinguish them may obscure real differences between groups. Further, whether the duration or severity of an exposure also modulates its penetrance to the next generation should be assessed. Methodological advances, particularly techniques for deep profiling of epigenetic marks in individual sperm and preimplantation embryos, will be instrumental in resolving these questions and advancing our understanding of how environmental information is inherited across generations.

## 7. Conclusions

Sperm passes more than just DNA to the embryo as they also carry epigenetic information that can instruct embryonic development. Environmental exposures can modify the sperm epigenome, mediating the transfer of exposure-induced information from father to offspring. These inherited changes can manifest in the preimplantation embryo and influence development, potentially leading to altered phenotypes in the next generation. Although various epigenetic features have been proposed as vectors of IEI, the interaction of all epigenetic layers and how they are inherited remains to be better understood. Continued research into sperm-to-embryo epigenetic transfer is needed to understand how environmental information is passed between generations.

## 8. Future perspective

In the next 5–10 years, research on IEI will likely move beyond descriptive associations and toward mechanistic understanding. Advances in single-cell multi-omics, allele-specific analyses and precise genome and epigenome editing tools will enable researchers to dissect how specific epigenetic features in sperm interact to instruct embryonic development. A particular focus may be placed on resolving whether and how these epigenetic marks are retained, remodeled or reestablished after fertilization. Emerging models that combine genetic engineering with environmental exposures shall further clarify causal relationships. In parallel, efforts to quantify and model penetrance and variability of inherited phenotypes will contribute to understand why only subsets of offspring are affected by paternal exposure. Ultimately, these developments will reveal how the effects of environmental experiences are transmitted across generations and how negative impacts of parental environments can be prevented or reversed.

## Article highlights

- The sperm epigenome is highly specialized and can be altered by environmental exposures.
- Sperm-derived epigenetic information instructs transcription and development of the preimplantation embryo.
- Paternal environmental exposures modify the sperm epigenome and can influence embryo gene expression and developmental trajectories.
- Inherited effects of environmental exposures can show incomplete penetrance, potentially due to variability in sperm epigenetic alterations or embryonic responses.
